# Exploring the effects of combined Nordic and reverse Nordic hamstring exercises on physical fitness in prepubertal male soccer players

**DOI:** 10.3389/fspor.2026.1717594

**Published:** 2026-02-27

**Authors:** Senda Sammoud, Yassine Negra, Raja Bouguezzi, Aaron Uthoff, Jason Moran, Helmi Chaabene

**Affiliations:** 1 Research Laboratory (LR23JS01) “Sport Performance, Health & Society”, Higher Institute of Sport and Physical Education of Ksar Saïd, University of La Manouba, Manouba, Tunisia; 2 Institut Supérieur de Sport et de l’Education Physique du Kef, Université de Jendouba, Le Kef, Tunisia; 3 AUT Millennium, School of Sport and Recreation, Sports Performance Research Institute New Zealand (SPRINZ), Auckland, New Zealand; 4 School of Sport, Rehabilitation, and Exercise Sciences, University of Essex, Colchester, United Kingdom; 5 Department of Cardiology and Angiology, University Hospital Magdeburg, Magdeburg, Germany

**Keywords:** athletic performance, biological age, eccentric training, team sports, youth development

## Abstract

**Introduction:**

This study aimed to examine the effects of combined Nordic Hamstring and Reverse Nordic exercises on measures of physical fitness in prepubertal male soccer players.

**Methods:**

The eccentric training program lasted 8 weeks, with two sessions per week, each lasting approximately 20 min and performed immediately after the warm-up of regular soccer training. A total of 34 prepubertal male soccer players were recruited and randomly assigned at the group level to either the eccentric training group (*n* = 17; age = 12.44 ± 0.24 years, maturity offset = −1.27 ± 0.28) or the active control group (*n* = 17; age = 12.40 ± 0.22 years, maturity offset = −1.15 ± 0.35). Physical fitness was assessed through measures of linear sprint performance (10 & 20 m), change of direction (505 test), agility (Y-shaped agility test), and vertical (countermovement jump) and horizontal jump performance (standing long jump).

**Results and discussion:**

The findings showed moderate group-by-time interactions for all fitness measures [effect size (d) = 0.62–1.16]. *Post hoc* analyses revealed small-to-moderate improvements in the eccentric training group across all performance measures (d = 0.31–1.18), while no significant changes were observed in the control group for any fitness measure. In conclusion, our findings support the effectiveness, practicality, and safety (no injuries reported) of the eccentric training program for improving physical fitness in prepubertal male soccer players.

## Introduction

Sprinting, jumping, change of direction (CoD) speed, and agility are key athletic qualities that significantly influence soccer performance (1, 41). These attributes are crucial for match performance, especially among young soccer players (2, 3). Although these qualities naturally develop with growth and maturation in youth athletes (4), they can be further enhanced with focused, sport-specific training programs (5). Therefore, it is imperative to develop and implement both appropriate and well-structured training interventions that address the key fitness demands of male youth soccer players in competition to achieve sporting success in their future careers.

Among the various training methods available, eccentric training (ET) has received increasing attention due to its positive effects on physical fitness in young athletes (6, 7). While much of the evidence originates from adult populations, research in youth athletes indicates that ET can be safely implemented when appropriately supervised and progressively overloaded (6, 8). Notably, ET elicits meaningful neuromuscular adaptations while limiting excessive mechanical stress (6, 8). Several studies have shown that ET is more effective than concentric-only or traditional training for improving muscle mass, muscle power, sprint speed, and stretch-shortening cycle performance in youth (6, 8–11).

Specifically, improving the eccentric strength of the knee flexors, particularly the hamstrings, is crucial for both performance enhancement and injury prevention in team-sport players (12, 13). Eccentric knee-flexor strength has been strongly associated with knee stability, which is a key determinant of CoD performance (30). In addition, knee-flexors contribute substantially to horizontal force production during sprinting through hip extension (14). Similarly, knee extensors play a fundamental role in eccentric braking, force absorption, and re-acceleration during sprinting and CoD tasks, thereby supporting performance and protecting the knee joint (15, 16). The Nordic Hamstring Exercise (NHE) is a popular eccentric exercise for strengthening the knee flexors and has been shown to improve physical fitness in sports such as soccer, handball, and tennis (17, 18). Additionally, the Reverse Nordic Exercise (RNE) has emerged as a simple and practical exercise targeting the knee extensors. Mounting evidence indicated promising improvements in physical fitness following RNE training in youth (19, 20) and young adults (21, 22).

To date, only two studies have examined the effects of RNE training in youth athletes (19, 20) highlighting the need for further investigation. Notably, no study has examined the combined effects of NHE and RNE within a single training program in youth soccer players. Therefore, this study aimed to examine the effects of an eight-week training program combining NHE and RNE on measures of physical fitness in prepubertal male soccer players. We hypothesized that integrating NHE and RNE into the standard soccer training routine would result in greater improvements in physical fitness outcomes compared with regular soccer training alone.

## Methods

### Participants

With reference to a recent similar study by Oueslati et al. (20), an *a priori* power analysis, with a type I error rate of 0.05% and 80% statistical power was computed. The analysis indicated that a total of 24 participants would be sufficient to observe a significant interaction effect (effect size Cohen's *d* = 0.98 for the 505 CoD test). To account for potential participant attrition, a total of 34 soccer players from two different regional soccer teams were allocated to either the ET group (*n* = 17) or the active CG (*n* = 17). Group allocation was performed at the team level rather than at the individual level. Specifically, one regional soccer team was randomly assigned to the ET group, while the second regional soccer team was assigned to the active control group. Therefore, this study should be considered a cluster-controlled trial rather than an individually randomized controlled trial. This approach was adopted to avoid contamination between interventions within the same team environment. To be included in the study, participants had to be in good health and free from musculotendinous injuries over the last six months preceding the study. Additionally, players who missed more than 20% of the total training sessions or two consecutive training sessions were excluded from the final analysis. Table 1 presents the anthropometric data for both groups. The maturity offset (MO) method was used to assess the biological maturity of participants (42). The following prediction equations were applied:

MO=−7.999994+(0.0036124*age*height).

**Table 1:** Anthropometric characteristics of the included participants.

Parameters	ET group (*n* = 17)	CG (*n* = 17)
Age (years)	12.44 ± 0.24	12.40 ± 0.22
Body height (cm)	147.71 ± 5.52	152.98 ± 6.47
Body mass (kg)	38.17 ± 3.24	40.60 ± 4.96
Maturity offset (years)^a^	−1.27 ± 0.28	−1.15 ± 0.35
APHV	13.71 ± 0.28	13.54 ± 028

Data are presented as means and standard deviations; ET, eccentric training group; CG, control group; APHV, age at peak height velocity.

^a^ As years from peak height velocity. .

All the experimental procedures and the associated potential risks were fully explained and written informed consent (parents/legal guardians) and assent (participants) were obtained before the commencement of the study. All procedures were approved by the local Institutional Review Committee of the ***blinded for review*** (approval number: 301k035, 22 January 2024), and conducted per the latest version of the Declaration of Helsinki.

### Experimental approach to the problem

This study aimed to examine the effects of an ET program, focusing on the NHE and RNE, on physical fitness measures in prepubertal male soccer players. Two regional teams of prepubertal male soccer players were assigned to either an ET group or an active control group (CG). Both groups participated in five soccer training sessions per week. The ET group substituted 10–20 min of low-intensity soccer drills with eccentric exercises on Tuesdays and Thursdays, while continuing their regular soccer-specific training afterward. During the same time period, the CG followed the team's usual training schedule and replaced the experimental intervention with standard soccer-specific technical and tactical drills, including passing sequences, positional play, and small-sided games. These drills were performed at a moderate intensity consistent with the team's habitual training structure and were matched in duration to the ET sessions. Prior to baseline testing, two familiarization sessions were conducted to help participants acclimate to the testing procedures. A variety of physical fitness tests were used to assess changes before and after the ET program, including linear sprint (10- and 20-m sprints), CoD (505 COD test), agility (Y-shaped agility test), jump height (countermovement jump [CMJ), and jump distance (standing long jump [SLJ). Testing occurred over two days with linear sprint, CoD, and agility testing conducted on the first day, jump testing on the second day. All tests were scheduled at least 48 h after the last training session or match and were conducted at the same time of day (18:00–19:30).

### Linear sprint time

Ten-meter linear sprint performance was assessed using a single-beam electronic timing system (Wittygate, Microgate, SRL, Bolzano, Italy). Participants started in a standing split stance position with their lead foot 0.3 m behind the first infrared photoelectric gate, which was placed 0.75 m above the ground to ensure that it captured trunk movement and avoided false signals through limb motion. In total, three single-beam photoelectric gates were placed at the start (0 m), middle (10 m), and finish (20 m) in order to quantify 0–10 m and 0–20 m sprint times. No rocking or false steps were permitted before starting. The between-trial recovery time was three minutes. The best performance out of two trials was used for further analysis. The between-trials intraclass correlation coefficients (ICCs) _(3,1)_ and the standard error of measurements (SEMs) were 0.81, 0.85 and 2.16, 2,40%, for the 5-, and 10-m sprint test, respectively.

### The 505 change of direction

The 505 CoD speed test was administered as per the protocol previously outlined by Negra et al. (23), using an electronic timing system (Wittygate, Microgate, SRL, Bolzano, Italy). Players assumed a standing position 10 m from the start line, sprinted as quickly as possible through the start/finish line, pivoted 180° at the 15-m line indicated by a cone marker, and returned as fast as possible through the start/finish line. To ensure proper execution of the test, a researcher was positioned at the turning line. If the participant changed direction before reaching the turning point, the trial was disregarded and reattempted after a three-minute recovery period. A between-trial rest period of three minutes was provided. The best performance out of two trials was used for further analysis. The between-trials ICC _(3,1)_ and the SEM were 0.94 and 1.23%.

### Y-shaped agility test

The Y-shaped agility test was administered using the protocol as previously outlined by Lockie et al. (24). The Witty light-based timing system was used to record the time and set the reactive conditions. The width of the gates was 1.5 m with a height of 1.2 m. The participants began 0.3 m behind the start line and ran maximally in a 5 m straight sprint. Then they performed a 45° change of direction to the left or to the right side in response to a stimulus, followed by a 5 m-long sprint to the finish gates. As a stimulus, a green arrow was used to dictate directional change. It appeared with a delay of approximately 40–45 ms after passing the starting gate. Two trials were performed, and the best time was taken for further analysis. A rest period of three minutes was allowed between trials. The between-trials ICC _(3,1)_ and the SEM were 0.85 and 1.33%.

### Countermovement jump

During the CMJ, participants started from a standing position and performed a fast downward movement by flexing the knees and hips before rapidly extending them to perform a maximal vertical jump (43). During the test, participants were instructed to maintain their arms akimbo. Jump height was recorded using an optoelectric system (Optojump next, Microgate, SRL, Bolzano, Italy). A rest period of 3 min was allowed between trials. The best out of two trials was retained for further analysis. Each participant performed two CMJ trials, separated by a three-minute rest period. The best trial was retained for further analysis, as it was considered to best reflect maximal vertical jump performance and to minimize the influence of sub-maximal or technically sub-optimal attempts. The between-trials ICC _(3,1)_ and the SEM were 0.84 and 3.23%.

### Standing long jump

The standing long jump (SLJ) was performed according to the protocol described by Marković et al. (43). Participants jumped forward with maximal effort from a standing position using a countermovement and arm swing. The horizontal distance from the starting line to the heel of the rear foot was measured to the nearest 1 cm using a tape measure. Two trials were performed with a 3-minute rest interval, and the best trial was retained for analysis. Reliability was acceptable [ICC_(3,1)_ = 0.89; SEM = 2.75%].

### Eccentric training program

The ET program, detailed in Table 2, included two exercises: the NHE and the RNE. Training was conducted twice per week during the first half of the in-season period (November–December 2024). Each session began with a 2–3-min warm-up including jogging and dynamic movements (e.g., high knees, butt kicks), followed by 2–3 min of dynamic stretching (e.g., hip flexor stretches, leg swings, arm circles). The first weekly session was scheduled at least 48 h after the last weekend soccer match, and the second session 48 h later (typically Tuesday and Thursday). During weeks 1–2, participants completed 2 sets of 4–6 repetitions per exercise, focusing on technique and familiarization. In weeks 3–4, volume increased to 3 sets of 6–8 repetitions, with reduced assistance for NHE and a greater range of motion for RNE. Weeks 5–6 involved 3 sets of 8–10 repetitions, emphasizing maximal eccentric strength with minimal assistance. During the final two weeks, participants performed 3 sets of 10–12 repetitions per exercise, aiming for full control and maximal range of motion. Rest periods of 60–90 s separated each set, and the total session duration was ∼15–20 min, including warm-up.

**Table 2:** Eccentric training program.

Week	Aim	Exercise	Recommendations	Sets	Reps/set	Rest between sets/exercise (s)
1	Familiarisation and technique focus	NHE	NHE: Focus on a slow, controlled descent and use hand support assistance during the initial weeks to avoid overloading. RNE: Ensure smooth, controlled movement with emphasis on knee and hip alignment.	2	4–6	60–90
		RNE		2	4–6	60–90
2		NHE		2	4–6	60–90
		RNE		2	4–6	60–90
3	Progression in Volume and Intensity	NHE	NHE: Gradually reduce assistance. RNE: Maintain control and form while progressively increasing the range of motion	3	6–8	60–90
		RNE		3	6–8	60–90
4		NHE		3	6–8	60–90
		RNE		3	6–8	60–90
5	Strength Emphasis	NHE	NHE: Focus on minimizing assistance during the eccentric phase RNE: Increase the range of motion while maintaining proper knee tracking	3	8–10	60–90
		RNE		3	8–10	60–90
6		NHE		3	8–10	60–90
		RNE		3	8–10	60–90
7	Maximal Effort	NHE	NHE: Encourage full control, aiming for complete independence by the final week RNE: Full range of motion with good technique	3	10–12	60–90
		RNE		3	10–12	60–90
8		NHE		3	10–12	60–90
		RNE		3	10–12	60–90

NHE, Nordic hamstring exercise; RNE, reverse Nordic exercise, Reps, repetitions.

Safety was monitored through participant self-report. Participants were instructed to immediately report any pain, discomfort, or other adverse sensations related to the exercises. No formal injury surveillance system was implemented. All sessions were supervised by coaching staff to ensure proper execution and adherence to the protocol.

## Statistical analyses

All data analyses were performed using SPSS 25.0 (SPSS, Inc., Chicago, IL, USA). Data are presented as means and standard deviations (SD). The normality assumption was tested and confirmed using the Shapiro–Wilk test. To establish the effect of the interventions on the dependent variables, a 2 (group: ET and CG) × 2 (time: pre, post) ANOVA with repeated measures was determined for each parameter. When group × time interactions reached the level of significance (i.e., significant *F*-value), group-specific *post hoc* tests (i.e., paired *t*-tests) were used. To determine the magnitude of the training effect within and between groups, Cohen's d effect size (ES) statistics were calculated. According to Hopkins et al. (25), ES values are classified as trivial (<0.2), small (0.2–0.6), moderate (0.6–1.2), large (1.2–2.0), very large (2.0–4.0), and extremely large (>4.0). To control for multiple comparisons across dependent variables, *p*-values associated with the group × time interaction effects were adjusted using the Holm–Bonferroni correction. The alpha level of significance was set at *p* < 0.05.

## Results

All participants received the treatment as allocated, with no training or test-related injuries reported. Physical fitness measures at baseline and follow-up are presented in Table 3. At baseline, there were no significant between-group differences in anthropometric characteristics or MO. All participants were rated as pubertal (Table 1). Similarly, no between-group differences were observed at baseline for any measure of physical fitness (Table 3).

**Table 3:** Group-specific changes in measures of physical fitness from pre-to-post training.

Testing measure	ET (*n* = 17)		Post-pre difference (95% CI)	CG (*n* = 17)		Post-pre difference (95% CI)	ANOVA	
	Pretest	Posttest		Pretest	Posttest		*p*-value (*d*)	
	M ± SD	M ± SD		M ± SD	M ± SD		Time	Group × Time
10-m sprint (s)	2.13 ± 0.08	2.09 ± 0.07	−2.24 [−3.91 to −0.56]	2.06 ± 0.07	2.08 ± 0.10	0.88 [−0.78–2.54]	>0.05 (0.4)	<0.05* (0.88)
20-m sprint (s)	3.79 ± 0.20	3.68 ± 0.16	3.24 [−5.58 to −0.89]	3.61 ± 0.15	3.63 ± 0.20	0.62 [−1.07–2.30]	>0.05 (061)	<0.05* (0.85)
505-CoD speed test (s)	2.73 ± 0.17	2.60 ± 0.09	−4.87 [−7.30 to −2.44]	2.66 ± 0.13	2.62 ± 0.13	−1.55 [−3.23–0.14]	<0.001 (1.43)	<0.05* (0.73)
Y-shaped agility test (s)	3.25 ± 0.31	2.95 ± 0.19	−9.19 [−13.68 to −4.70]	3.06 ± 0.16	3.03 ± 0.12	−0.94 [−3.13–1.25]	<0.01 (1.35)	<0.01* (1.1)
CMJ (cm)	21.02 ± 3.94	22.45 ± 5.14	6.53 [0.93–12.13]	21.80 ± 4.20	21.60 ± 4.03	−0.90 [−6.64–4.83]	>0.05 (0.58)	<0.01*(0.62)
SLJ (m)	1.66 ± 0.13	1.76 ± 0.17	6.39 [2.56–10.22]	1.66 ± 0.16	1.64 ± 0.20	−1.31 [−3.36–1.01]	<0.05 (0.79)	<0.05* (1.16)

M, mean; SD, standard deviation; CI, confidence interval; d, Cohen's d; ET, eccentric training; CG, control group; SLJ, standing long jump; CMJ, countermovement jump; CoD, change of direction.

* Still significant after Holm–Bonferroni correction.

### Linear sprint-time

Findings indicated significant group × time interaction for the 10- (*d* = 1.27 [large], *p* < 0.001, and the 20-m [*d* = 1.24 (large), *p* < 0.01] sprint test (Table 3). *Post hoc* analyses showed a moderate pre-to-post performance improvement in the ET group for the 10-m [Δ-2.24%; *p* < 0.05; *d* = −0.67 95% CI 95% (0.72–0.77)], and 20-m [Δ-3.24%; *p* < 0.05; *d* = 0.75, 95% CI (0.65–0.81)] linear sprint performance. However, the active CG did not show any significant improvement for the 10-m [Δ-0.88%; *p* > 0.05; *d* = 0.24 (small), 95% CI (0.19–0.27)] and the 20-m [Δ-0.62%; *p* > 0.05; *d* = 0.12 (trivial), 95% CI (0.02–0.19)] linear sprint performance.

### Change of direction speed

The results indicated a significant group × time interaction [*d* = −0.75 (moderate), *p* < 0.05] (Table 3). *Post hoc* analyses revealed a moderate improvement in 505 CoD times from pre-to-post for the ET group [Δ-4.87%; *p* < 0.01; *d* = 0.98, 95% CI (0.90–1.03)]. No significant pre-to-post changes were observed for the CG [Δ-1.54%; *p* > 0.05; *d* = 0.32 (small), CI 95% (0.26–0.38)].

### Y-shaped agility test

The finding showed a significant group × time interaction [*d* = 1.11 (moderate), *p* < 0.01] (Table 3). *Post hoc* analyses demonstrated a large performance improvement from pre-to-post for the ET group [Δ-9.19%; *d* = −1.20, 95% CI (1.06–1.29); *p* < 0.01]. No significant pre-to-post changes were observed for the CG [Δ-0.94%; *p* > 0.05; *d* = 0.22 (small), 95% CI (0.14–0.28)].

### Jumping performance

For CMJ, a tendency forward a significant group × time interaction [*d* = 0.71 (moderate), *p* = 0.05] was observed (Table 3). *Post hoc* analyses demonstrated a small CMJ-height improvement for the ET group [Δ6.53%; *p* < 0.05; *d* = 0.23, 95% CI (0.20–0.25)]. The CG revealed no significant pre-to-post change [Δ-0.90%; *p* > 0.05; *d* = 0.05 (trivial), 95% CI (0.03–0.07)]. For the SLJ test, a significant group × time interaction [*d* = 1.23 (large), *p* < 0.01] was noted. *Post hoc* analyses indicated a moderate pre-to-post enhancement for the ET group [Δ6.39%; *d* = 0.68, 95% CI (0.60–0.74); *p* < 0.01]. However, the CG did not reveal any significant change [Δ-1.31%; *p* > 0.05; *d* = 0.11 (trivial), 95% CI (0.04–0.21)].

## Discussion

The goal of this study was to explore the effects of an eight-week, NHE- and RNE-based ET program on physical fitness in prepubertal male soccer players. The main findings revealed that the ET program induced small-to-large improvements in sprint speed, jumping, CoD speed, as well as agility in prepubertal male soccer players, whereas players who followed regular soccer training alone showed no significant improvements. These results suggest that integrating NHE and RNE into regular soccer training can enhance key athletic qualities relevant to soccer performance. Given the importance of these attributes, ET may be a valuable addition to youth development programmes. However, these findings should be interpreted within the specific context of prepubertal athletes whose neuromuscular and hormonal profiles differ from those of postpubertal or adult populations and may influence training adaptations.

Linear sprint performance over short distances is a critical fitness attribute for soccer players, enabling to accelerate rapidly, outpace opponents, and respond to dynamic match situations (26). In the present study, the ET group significantly improved both 10-m and 20-m sprint performance with moderate effect sizes, while the CG did not show any significant changes. Our findings align with previous research demonstrating the effectiveness of ET for improving sprint performance. Although direct strength or neuromuscular measurements were not conducted, the observed improvements may be partly explained by enhanced force production and neuromuscular coordination commonly associated with ET (9, 19). There is evidence that postpubertal athletes or those exposed to higher eccentric loading may exhibit greater responsiveness than prepubertal soccer players (27). As such, it is possible that factors such as maturation status and training volume and/or intensity could influence responsiveness to training. Overall, these results suggest that regular soccer training alone may be insufficient to elicit meaningful improvements in short-distance sprint performance in prepubertal male soccer players.

Change of direction speed and agility are crucial fitness attributes for soccer performance, allowing rapid responses in an unpredictable game environment (28). The ET group demonstrated moderate improvements in the 505 CoD speed test and large improvements in the agility test, whereas no significant changes were observed in the CG. These results support previous evidence highlighting the effectiveness of ET for enhancing CoD and agility performance (19, 29). However, comparisons with previous studies should be made cautiously, as differences in sport-specific demands, movement patterns, sex, biological maturation, and baseline strength may influence outcomes. The observed improvements may be attributed to enhanced eccentric strength of the knee extensor and flexor, which play a critical role during deceleration and re-acceleration phases of CoD speed and agility tasks (30, 31). Although these mechanisms were not directly assessed, the moderate-to-large effect sizes align with previous findings showing that ET improves braking capacity, joint stability, and movement control (9, 32). Nevertheless, these interpretations remain speculative and should be confirmed by future studies incorporating neuromuscular and biomechanical assessments.

Jumping ability is a key physical requirement in soccer, particularly in aerial duels, heading actions, and defensive clearances (28, 44). In the present study, the ET group showed small-to-moderate improvements in CMJ-height and SLJ-distance, while no significant changes were observed in the CG. These findings are consistent with previous research reporting improvements in jump performance following ET interventions (19, 23, 29). The relatively smaller effect sizes observed in the present study may be partly explained by the prepubertal status of the participants, as neuromuscular and musculotendinous adaptations to eccentric loading are typically less pronounced before puberty (33, 34). Additionally, differences in training content and the emphasis on horizontal vs. vertical force production may influence jumping outcomes (35). The improvements observed in CMJ-height and SLJ-distance performances may reflect enhanced stretch-shortening cycle efficiency, a known adaptation to ET (11, 45). Given the frequent explosive and jumping actions required in soccer, incorporating eccentric exercises such as NHE and RNE may provide an effective strategy to improve lower-body power in prepubertal male soccer players.

### Limitations

This study has some limitations that readers should consider. First, participants were allocated by team rather than individual randomization, which introduces potential clustering and selection bias. This limitation may weaken causal inference, and future studies should consider using randomized individual allocation. However, the limited number of teams did not allow for robust multilevel or mixed-effects modeling to formally account for clustering effects; therefore, the findings should be interpreted with caution as evidence derived from a real-world applied setting rather than definitive causal conclusions. Second, while the overall exposure to training was comparable between the experimental training (ET) and control groups (CG), it would have been beneficial to track the training load throughout the eight weeks using both external (e.g., total distance covered) and internal (e.g., rate of perceived exertion, heart rate) metrics. Third, although strength adaptations were suggested to play a role in performance improvements, direct measurements of eccentric muscle strength were not conducted. Future studies should address this gap. Fourth, we did not incorporate direct physiological (e.g., electromyography) or biomechanical (e.g., vertical ground reaction force) measures in this study. Fifth, while the study design allows for the investigation of the combined effects of Nordic and Reverse Nordic exercises, it does not permit the identification of the individual effects of each exercise, an aspect that should be addressed in future studies. Sixth, the *a priori* power analysis was based on a single primary outcome (505 CoD test). While this is common practice, it may not fully reflect the power for other outcomes, which should be considered when interpreting the results. Finally, our sample consisted solely of prepubertal male soccer players from two regional teams; therefore, caution should be exercised when generalizing these findings to broader youth populations, female athletes, or players from different competitive levels. Including these assessments in future research could offer more comprehensive insights into the neuromuscular and biomechanical mechanisms at play in ET and its impact on athletic performance.

### Conclusions and practical applications

This study showed that an eight-week ET programme based on both the NHE and RNE, integrated twice per week into regular soccer training, was effective in enhancing linear sprint performance, CoD speed, jumping ability, and agility in prepubertal male soccer players. However, regular soccer training alone did not elicit any significant adaptations. Additionally, the programme was well-tolerated and easily integrated with no injuries reported throughout the training period, reinforcing its practicality and safety. Therefore, coaches and practitioners are advised to consider implementing NHE and RNE as part of structured in-season training to enhance key physical fitness components of prepubertal male soccer players.

## Data Availability

The raw data supporting the conclusions of this article will be made available by the authors, without undue reservation.
